# Epigenetic regulation of autophagy in neuroinflammation and synaptic plasticity

**DOI:** 10.3389/fimmu.2024.1322842

**Published:** 2024-02-22

**Authors:** Isaac Bai, Cameron Keyser, Ziyan Zhang, Breandan Rosolia, Jee-Yeon Hwang, R. Suzanne Zukin, Jingqi Yan

**Affiliations:** ^1^ Center for Gene Regulation in Health and Disease, Department of Biological, Geological, and Environmental Sciences, Cleveland State University, Cleveland, OH, United States; ^2^ Department of Pharmacology and Neuroscience, Creighton University School of Medicine, Omaha, NE, United States; ^3^ Dominick P. Purpura Department of Neuroscience, Albert Einstein College of Medicine, New York, NY, United States

**Keywords:** autophagy, epigenetics, neuroinflammation, microglia, synapse

## Abstract

Autophagy is a conserved cellular mechanism that enables the degradation and recycling of cellular organelles and proteins *via* the lysosomal pathway. In neurodevelopment and maintenance of neuronal homeostasis, autophagy is required to regulate presynaptic functions, synapse remodeling, and synaptic plasticity. Deficiency of autophagy has been shown to underlie the synaptic and behavioral deficits of many neurological diseases such as autism, psychiatric diseases, and neurodegenerative disorders. Recent evidence reveals that dysregulated autophagy plays an important role in the initiation and progression of neuroinflammation, a common pathological feature in many neurological disorders leading to defective synaptic morphology and plasticity. In this review, we will discuss the regulation of autophagy and its effects on synapses and neuroinflammation, with emphasis on how autophagy is regulated by epigenetic mechanisms under healthy and diseased conditions.

## 1 Introduction

Autophagy is a conserved cellular mechanism that enables the degradation of cellular components *via* the lysosomal pathway (1–6). This process can be triggered by various cellular signals including hypoxia, nutrient deficiency, and pathogens (5–9). Accumulating evidence indicates that autophagy is associated with many brain-related disorders (10–18). In neurons, autophagy is increased under the conditions of low neuronal activity, and loss of neurotrophic factors which is induced indirectly *via* inhibiting the mammalian target of rapamycin (mTOR) signaling in response to the starvation of amino acids (1, 2, 12, 13, 15, 16). There are three primary and distinct forms of autophagy in mammalian cells: macroautophagy, microautophagy, and chaperone-mediated autophagy (CMA) (1, 2, 4–6, 13, 19). In this review, we focus on macroautophagy (hereafter called autophagy), which is the main form of autophagy used by neurons to maintain cellular homeostasis, control the quality of proteins, and regulate synaptic plasticity (10, 20–22). In neurons, autophagy has been observed to occur compartmentally within the soma, axons, dendrites, and synapses (1, 23), and is involved in presynaptic function, synapse elimination, and synaptic plasticity (12, 24). The neuroanatomical hallmark of autophagy is the presence of autophagosomes, which are double-membrane vesicles that sequester cytoplasmic components for subsequent degradation by the lysosomes (10, 25). Autophagosomes utilize cargo adapters such as p62 and NBR1 to bind and deliver proteins and organelles for lysosomal degradation (26–28). These adapters can recognize and bind cargos such as ubiquitinated proteins, and connect cargos to autophagic machinery, thus enabling their engulfment by autophagosomes targeted for degradation (23, 26). In mice, deficient autophagy in microglia and neurons leads to impairment of synaptic pruning, resulting in increased numbers of immature synapses, correlating with social deficits in autism spectrum disorders (ASDs) (15, 16, 29).

Autophagy is regulated by different upstream signal pathways (5–9, 30, 31). mTOR is a serine/threonine kinase that is a regulator of cellular metabolism and plays a central role as a negative regulator of autophagy (32–34). Autophagosome formation is initiated by the activation of ULK1 (unc-51-like autophagy activating kinase 1), which drives the formation of the isolation membrane (phagophore), the precursor membrane structure of autophagosomes (35, 36). This process is facilitated *via* the direct activation of the VPS34 complex and mediating trafficking of *Atg9* (36, 37). In neurons, mTOR complex 1 (mTORC1) is localized to both presynaptic and postsynaptic sites where it inhibits autophagy (38, 39). mTORC1 acts as a negative regulator of autophagy by suppressing autophagosome formation *via* phosphorylation-dependent inhibition of ULK1/ULK2 at S757, a target of mTORC1 and well-known anti-autophagy site (19, 30). mTORC1 also suppresses the formation of the VPS34 complex, preventing the formation of phagophores (36, 40). In contrast, in response to cellular stressors, such as starvation or hypoxia, the adenosine monophosphate-activated protein kinase (AMPK) pathway is activated (30, 41–43). The activated AMPK inhibits mTORC1 directly by phosphorylation of the mTORC1 subunit Raptor at Ser-792, which inhibits mTORC1 kinase activity (44). AMPK also can indirectly inhibit mTORC1 by phosphorylation of the regulator protein TSC2 at S1387, which reduces the contact of mTORC1 with its activator Rheb-GTP (43, 45). Furthermore, AMPK acts as a positive regulator of autophagy by directly phosphorylating and activating ULK1 at S317 (30). This phosphorylation increases the activity of ULK1, promoting the phosphorylation of *Atg6* (*beclin1* in mammals) at S14, a crucial step in the nucleation phase of autophagy, and recruits the PI3K complex, which is needed for the elongation of the phagophore (37). Phosphorylated Beclin1 promotes lipidation of LC3-I (*Atg8*) to generate its lipidated form LC3-II, which enables elongation of the membrane and formation of mature autophagosomes (1, 46). The forming autophagosomes then act *via* cargo adaptor proteins (such as p62) to engulf protein aggregates, compromised organelles, and ubiquitinated proteins. Mature autophagosomes fuse with lysosomes and become autolysosomes, which degrade cytosolic cargos inside (1, 2, 23, 46).

This review summarizes current knowledge on the role autophagy plays in the regulation of synaptic development/functions and the epigenetic regulation of autophagy. Aberrant epigenetic changes leading to autophagy dysregulation and the associated neuroinflammation in neurological diseases has also been discussed (**Table 1**).

**Table 1:** Dysregulated autophagy in different diseases.

Diseases	Autophagy	Epigenetic Regulation	Key Players
Alzheimer’s Disease (47, 48)	Downregulated	Unclear	LC3-II, LAMP1, p62
Huntington’s Disease (49)	Downregulated	Unclear	DNMT1
Parkinson’s Disease(50–52)	Downregulated	DNA Methylation	ULK1, SIRT1
Fragile X Syndrome (15, 53)	Downregulated	Unclear	mTORC1
Multiple Sclerosis (54-57)	Dysregulated	Unclear	ATG5, ATG16L2, ULK1
Tuberous Sclerosis Complex (19, 58)	Downregulated	Unclear	TSC1, TSC2, mTOR
Danon Disease (59)	Downregulated	DNA Methylation	LAMP2
Glioblastoma (60)	Downregulated	DNA Methylation	ULK2
Breast Cancer (61)	Downregulated	DNA Methylation	Beclin1

## 2 Autophagic regulation of synapse

**Figure 1:**
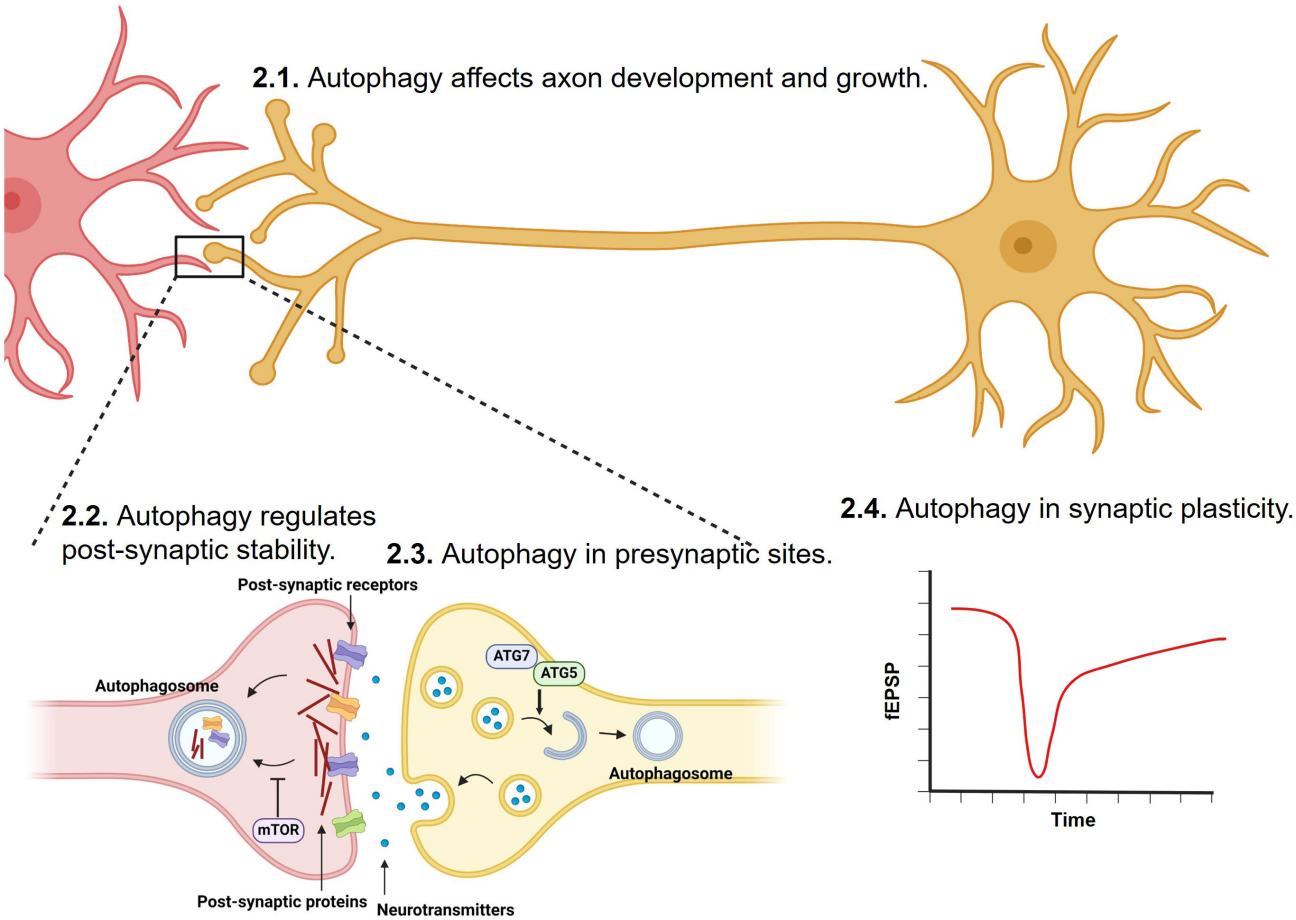
Regulation of synapse development, transmission, stability and plasticity by autophagy. Synaptic formation, strengthening, and elimination during development and adulthood are critical to healthy nervous system functions. Through degrading misfolded proteins, damaged organelles, post-synaptic proteins, and excessive synaptic vesicles, autophagy has been shown to play important roles in axon, and at both pre-synaptic and post-synaptic sites.

Synaptic formation and maintenance during development are critical to healthy nervous system functions. Autophagy plays important roles in synaptic development and maturation and is important for the maintenance of neuronal functions by regulating the clearance of misfolded proteins, damaged organelles, and excessive synaptic vesicles (SVs) (62–65) (**Figure 1**).

### 2.1 Autophagy in axon and synaptic development

Studies using Drosophila and *C. elegans* models have shown that axonal autophagosomes are evolutionarily conserved and form in neurons *in vivo*, demonstrating the importance of autophagy-mediated degradation of unwanted proteins and organelles in the development of axons and synapses (66–68). Recent studies on mice have identified two key autophagy-associated regulators of axonal and synaptic development *in vivo*. Mice lacking Alfy (WDFY3), a scaffold protein that mediates cargo-selective autophagy, displayed severe defects in the formation of all major axon tracts in the central nervous system (CNS), including reduced corpus callosum (69). Studies on mice lacking AP-4E, a positive regulator of autophagosome formation, resulted in a thinner corpus callosum and failed axon extension (70, 71). In order for the healthy development of synapses, there must also be proper axon growth and extension (72). A study conducted on Drosophila larvae lacking *Atg*s showed a significant reduction in boutons at neuromuscular junctions (73). On the contrary, another study shows the absence of *Atg*s in the developing retinal neurons in Drosophila showed a significant increase in the number of synapses (58). The latter study elucidated an interesting mechanism that autophagy mediates synaptogenesis by mediating the protein levels of synaptic seeding factors Liprin-α and Syd-1 (58).

Furthermore, autophagy plays a crucial role in synaptic pruning in early development. Tuberous sclerosis complex (TSC), a developmental disorder characterized by intellectual disability, epilepsy, and the presence of cortical tubers, arises from mutations that inhibit the function of the *Tsc1* and *Tsc2* genes (74). TSC1 and TSC2 promote neuronal autophagy by inhibiting mTOR signaling (19, 74). Many studies have tried to identify changes in dendritic spine density in mice with loss of function in *Tsc1* and *Tsc2*. Hippocampal granule cells in a *Tsc1* ^+/-^ mouse model show no effect of *Tsc1* haploinsufficiency on spine density (75). However, increased spine density was observed in cerebellar Purkinje cells at 4 weeks old in Purkinje cell-specific *Tsc1* heterozygous mice and also in *Tsc1* fully mutant mice (76). Meanwhile, a reduction in spine density was observed in the apical dendrite of cortical neurons in a mouse model with neuron-specific *Tsc1* knockout, and rapamycin treatment slightly increased the spine density (77). A study conducted on *Tsc1* knockout mice by *Tang et al.* was able to corroborate the loss of neuronal autophagy with reduced synaptic pruning (16).

Autophagy also plays a key role in maintaining energy homeostasis and supply in neurons and synapses (78, 79). Autophagy prevents the accumulation of defective proteins and organelles in neurons by recycling them for nutrients (80). Furthermore, autophagy is upregulated in nutrient-deprived environments, allowing for the degradation of nonessential cellular components to use for energy (1, 2). In addition, proper mitochondrial function is needed to maintain neuronal integrity throughout development (47). The deficits of synaptic mitophagy, cargo-specific autophagy for eliminating dysfunctional mitochondria, induces synaptic degeneration in a mouse model of Alzheimer’s disease (AD) (81). To support the high-energy demands of neurons, especially the pre-synaptic sites when releasing neurotransmitters, the efficient removal of damaged mitochondria through mitophagy is critical for the maintenance of energy homeostasis in neurons and synapses (82, 83). A recent study revealed that metabolically enhanced neurons exhibit extensive activation of mitophagy, as demonstrated by increased levels in LC3-II and LAMP1 (lysosomal-associated membrane protein 1), which facilitates mitochondrial turnover and sustains high-energetic activity in synapses (82).

### 2.2 Autophagy in post-synaptic sites

Most excitatory synapses in brains are located on dendritic spines, the postsynaptic protrusions that receive the varieties of excitatory input (84, 85). During early postnatal development, synapse formation exceeds synaptic pruning, resulting in an abundance of immature synapses (84, 85). The maturation of spines includes spine pruning/elimination and strengthening. During the critical period, spine pruning reduces the number of synapses, refining neural circuits that underly behaviors and cognition (16, 85). Aberrant spine morphology and increased percentage of immature spines have been described in autopsy brains of ASDs and many psychiatric diseases (86). In Fragile X syndrome, the most consistent anatomical finding in brains is an abnormal morphology of dendritic spines (87). Recent studies provide evidence of impaired autophagy as a potential mechanism underlying compromised synapse elimination, or an overabundance of dendritic spines in ASDs (15–17, 29). Particularly, in the postsynaptic spine site, autophagy is restricted during postnatal development in the neocortex of *Tsc2* ^-/-^ mice (16) and the hippocampus of *Fmr1* KO mice due to mTOR signaling overactivation (15). Furthermore, these findings revealed that downregulated autophagy leads to impaired spine pruning in cortical neurons and hippocampal neurons (16). Rapamycin, a mTORC1 inhibitor, has been demonstrated to improve spine pruning and social interactions in *Tsc2* ^-/-^ mice but not in mice with neuronal autophagy-deficient (*Atg7*cKO) or mice with *Tsc2* ^-/-^ and *Atg7*cKO double mutations (16). Moreover, *Atg7* knockout animals have ASD-like characteristics, such as poor social interactions and social novelty (16). In the hippocampus of *Fmr1* KO mice, the mouse model of Fragile X syndrome, genetic knockdown of *Raptor*, a defining component of mTORC1, restored the downregulated autophagy in CA1 neurons (15). mTORC1 activity is enhanced in hippocampal neurons from *Fmr1* KO mice, as the mice exhibited higher levels of phosphorylation of mTOR at S2448 (15). Expression of *shRNA* targeting *Raptor* reduced phosphorylation of ULK1 at S757 and increased the number of LC3+ autophagosomes (15). Expression of *shRaptor* activated autophagy, reduced spine density, and rescued the cognitive deficits of *Fmr1* KO mice (15). Mechanistically, activated autophagy degrades postsynaptic proteins to regulate synapse stabilization and plasticity in hippocampal neurons with *Fmr1* KO.

Autophagy has also been shown to be crucial for BDNF-induced synaptic plasticity and synapse remodeling (24). Fasting-induced BDNF signaling suppresses autophagy in mouse hippocampus, which may prevent the degradation of post-synaptic proteins such as PSD-95 and SHANK3. Suppression of autophagy restored the long-term potentiation (LTP) in hippocampal neurons with BDNF deficiency and contributed to the increased spine density induced by fasting (24). It also has been suggested that fasting increased BDNF-level to suppress autophagy, which promotes the formation of memory enhancement.

Furthermore, autophagy plays an important role in maintaining postsynaptic sites, as autophagy degrades unwanted or misfolded proteins to prevent their accumulation and subsequent aggregation (88). This degradation prevents the formation of amyloid-β (Aβ) oligomers or Tau fibrils, which impair synaptic function (88). For example, aggregates of Aβ in neurons are heavily involved in the early synaptic failure of AD pathogenesis, as Aβ oligomers bind to synaptic sites and reduce dendritic spine density, impairing LTP and memory capabilities (89). Without degradation *via* the autophagy/lysosomal pathway, protein aggregates also prevent the synaptic maturation and disrupt functions of neurons.

### 2.3 Autophagy in presynaptic sites

Furthermore, autophagy has been shown to play a key role in regulating SV turnover and neurotransmission (22, 53, 78). Unlike usual nutrient-deprived environments, synaptic autophagosome formation occurs relatively constantly regardless of changes of environmental condition and has been shown to mediate SV turnover (22, 90). A recent study using *Atg5* knockout mice, a crucial protein needed for autophagosome formation, resulted in increased excitatory neurotransmission in acute hippocampal slices (22). The authors detailed that the elevated neurotransmission was a result of elevated calcium release from endoplasmic reticulum (ER), as mice lacking *Atg5* show the selective accumulation of tubular ER in axons, implicating the interaction between autophagy and ER as a mediator for neurotransmission (22). Although impaired autophagy facilitates excitatory neurotransmission, several studies report opposite effects upon autophagy induction. A study on the role of synaptotagmin-7 (SYT7), a peripheral membrane protein that controls multiple modes of SV exocytosis and plasticity, revealed that mouse models with knockout of SYT7 exhibited much slower SV replenishment rate and fewer asynchronous releases of vesicles (48). In 2015, a study involving bacterial expulsion with knockdown of SYT7 with shRNA indicates that SYT7 is involved in lysosome exocytosis and autophagy (91). While specific mechanisms of SYT7 and SYT7/autophagy are not well-developed, upregulation of autophagy in neurons could promote SYT7 degradation, thus reducing SV turnover rate and exocytosis at the synapse. Furthermore, in mouse corticostriatal slices treated with rapamycin, autophagic vacuole-like structures appeared in axons and SVs decreased, indicating a link between induction of autophagy and decreases in SV turnover (53). The authors also observed that 8-week-old transgenic mice lacking *Atg7* expression in dopaminergic neurons show an abnormal enlargement of presynaptic terminals, which also released larger amounts of neurotransmitters and exhibited a faster presynaptic recovery (53).

### 2.4 Autophagy in synaptic plasticity

As autophagy extensively affects presynaptic neurotransmitter release, synapse development, and postsynaptic stability, it is not surprising that autophagy is crucial for the synapse plasticity. As discussed above, autophagy is important for the formation of LTP in mouse hippocampal neurons induced by fasting and BDNF (24). Rapamycin treatment could activate autophagy and significantly increase frequency and amplitude of mini excitatory postsynaptic currents (mEPSCs) in patient neurons (17). Past findings suggest that neuronal autophagy can facilitate the degradation of AMPA (α-amino-3-hydroxy-5-methyl-4-isoxazole propionic acid) and GABA (γ-Aminobutyric acid) receptors (92, 93). Autophagy was shown to be cell-autonomously needed in excitatory neurons for developmental dendritic spine pruning, a process thought to be mediated by processes akin to long-term depression (LTD) (94). LTD is a form of long-lasting synaptic plasticity that relies on the degradation of postsynaptic components and structures (62). While the specific mechanisms mediating LTD are not well characterized, a recent study reveals that autophagy is crucial for LTD (62). Using immunostaining, the authors confirmed the presence and formation of autophagic machinery in dendrites of cultured hippocampal cells after chemically induced LTD (62). They then demonstrated that dendritic autophagy was activated during LTD, as shown by increased levels of autophagic marker LC3-II and ULK1 complex components (62). Findings from analysis with phosphorylated ATG14 at S318, a direct target of ULK1, indicate that acute inhibition of autophagy is sufficient to abolish LTD (62). In AD models, additional Aβ aggregates from axons and dendrites have been shown to reduce local spine number and plasticity (89). Autophagy-mediated degradation of these aggregates facilitates synaptic plasticity (89). Taken together, these studies provide new insights into the role of autophagy in synaptic plasticity, again highlighting the potential therapeutic implications of manipulating autophagy as a novel solution to target autism and other neurological disorders.

## 3 Epigenetic regulation of autophagy

Epigenetic regulation refers to heritable changes in gene expression that are not caused by changes in the DNA sequence itself (95). These changes can be prompted by multiple factors such as environmental stimuli, diet, psychological stress, metabolic shifts, and cell signaling, and are mediated through various epigenetic mechanisms (95). In this section, we will focus on the role of epigenetic modifications on regulating autophagy. In epigenetic modification, histone proteins are subject to numerous post translational modifications including methylation, phosphorylation, ubiquitination, palmitoylation, dopaminylation, and acetylation (96). Acetylation is an important form of histone modification that mostly occurs at the N-terminus of H3 and H4 at lysine and is mainly modulated by histone acetyltransferases (HATs) and histone deacetylases (HDACs) (96, 97). HATs promote histone acetylation by catalyzing the transfer of an acetyl group to a lysine residue. Conversely, HDACs erase histone acetylation by catalyzing the removal of acetyl groups. Histone acetylation changes chromatin structure and thus regulates gene expression by making genes accessible or inaccessible to transcription factors (96). DNA methylation is an epigenetic mechanism in which a methyl group is transferred onto the cytosine to form 5-methylcytosine to repress gene expression (98). DNA methylation usually occurs on cytosine phosphate guanine (CpG) islands, a site where a cytosine is next to a guanine (98, 99). To form 5-methylcytosine, DNA methyltransferases (DNMTs) add a methyl group to the fifth carbon on cytosine located in CpGs (98, 99). DNA methylation blocks the binding of transcription factors or other regulatory proteins to DNA sequence (98). Accumulating evidence indicated that transcription is a critical regulatory step for autophagy. However, epigenetic regulation of neuronal autophagy genes has not been well understood. It is thus critical to bring more attention to study how autophagy genes are regulated by epigenetic mechanisms in brain under physiological and pathological conditions more in depth.

### 3.1 Regulation of autophagy by histone modification

**Figure 2:**
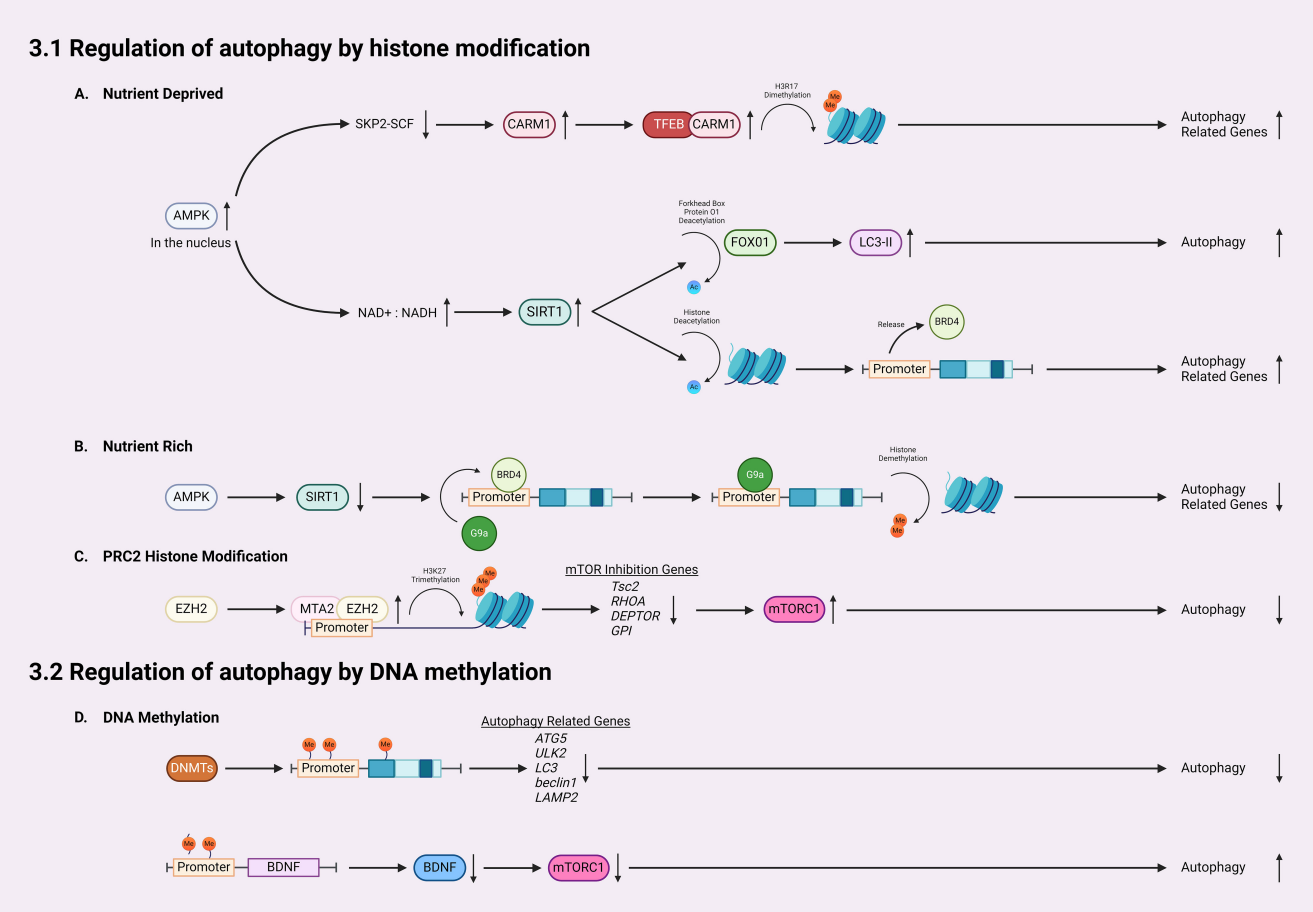
Mechanisms for epigenetic regulation of autophagy. **(A)** In nutrient-deprived conditions, AMPK accumulates in the nucleus, downregulating SKP2-SCF and thereby allowing the accumulation of CARM1. CARM1 acts as a coactivator of TFEB, leading to dimethylation on histone H3R17 and subsequent activation/expression of autophagy-related genes. Furthermore, following AMPK accumulation, the NAD+: NADH ratio increases, leading to SIRT1 activation which deacetylates histones, increasing LC3-II formation. **(B)** Activated SIRT1 also releases BRD4 from promoter regions of autophagic genes, allowing for increased expression of autophagy-related genes. In nutrient-rich conditions, SIRT1 remains inactivated, which allows BRD4 to bind the promoter regions and recruit G9a to promote histone demethylation and inhibit gene transcription. **(C)** EZH2 is recruited to the promoter region of autophagy-related genes by MTA2, catalyzing the trimethylation of lysine 27 of histone H3 thereby silencing the transcription of mTOR inhibition genes such as *Tsc2*, *RHOA, DEPTOR*, and *GPI*, which activates the mTOR pathway, inhibiting autophagy. **(D)** For regulation of autophagy by DNA methylation, DNMTs methylate promotors and coding regions of autophagy-related genes, silencing their transcription and therefore reducing autophagy. Finally, DNA methylation in the BDNF promoter region inhibits the expression of BDNF, lowering the activity of mTOR, and increasing autophagy.

In addition to activating autophagy through inhibiting mTORC1 activity, AMPK can influence HATs and HDACs by interfering with substrate availability or phosphorylating their cofactors (100). AMPK-mediated activation of SIRT1 (Sirtuin 1, a member of the sirtuin family and a class III HDAC) plays a crucial role in epigenetically regulating autophagy (100–103) (**Figure 2A**). Activation of AMPK increases the nicotinamide adenine dinucleotide (NAD+): NADH ratio, leading to increased activity of SIRT1 (100). Activated SIRT1 first induces autophagy *via* deacetylation of forkhead box protein O1 (FOXO1), which is normally upregulated following glucose deprivation, inducing increased LC3-II formation and elongation of phagophores (104). Secondly, AMPK-mediated SIRT1 activation could directly deacetylate histones to activate certain kinds of autophagy. Under nutrient-rich conditions, the histone H4 lysine 16 at promoter regions of certain autophagy and lysosomal-related genes is acetylated, which are recognized and bound by the epigenetic reader bromodomain-containing protein 4 (BRD4) (**Figure 2B**). Then, BRD4 recruits methyltransferase G9a to these regions, which represses gene expressions through dimethylation on histone H3K9. Thus, BRD4 and G9a work together to suppress autophagy and lysosomal biogenesis when AMPK/SIRT1 are not activated (103). However, under the nutrient-deprived conditions, AMPK-mediated SIRT1 activation directly deacetylates histone H4, which subsequently displaces BRD4 and G9a from the promoter, causing transcriptional activation of autophagy (103) (**Figures 2A, B**).

Transcriptional activation of autophagy is also linked with coactivator-associated arginine methyltransferase 1 (CARM1) (105, 106). A recent study reported a CARM1 mediated dimethylation in histone H3 Arg17 (H3R17me2) induced by glucose starvation, which triggered autophagy in mouse embryonic fibroblasts (MEFs) (106) (**Figure 2A**). Autophagic flux analysis revealed that glucose starvation induced LC3-II accumulation only occurred in wild-type MEFs but not CARM1 knockout MEFs (106). Importantly, overexpression of transcription factor EB (TFEB), a master activator of many autophagy/lysosomal genes, is unable to promote autophagy in CARM1 knockout MEFs, which demonstrates that CARM1-mediated dimethylation is critical for expressions of TFEB-targeted genes (106). In glucose-rich conditions, CARM1 is degraded by SKP2-SCF ubiquitin ligase (106). However, in glucose-deprived conditions, AMPK accumulates in the nucleus and phosphorylates FOXO3a, which suppresses SKP2 and stabilizes CARM1 to activate TFEB targeted autophagy genes (106). Thus, CARM1 serves as a co-activator of TFEB.

Histone methyltransferase EZH2 (enhancer of zeste homolog 2) is a subunit of polycomb repressive complex 2 (PRC2), a complex that catalyzes the trimethylation of lysine 27 on histone H3 (H3K27me3) to repress expression of genes *via* transcriptional silencing (107–111). EZH2 mediated trimethylation represses the expression of inhibitory regulators for the mTOR cascades, including *Tsc2, RHOA, DEPTOR*, and *GPI* (111) (**Figure 2C**). In doing so, EZH2 epigenetically activates mTORC1 and downregulates autophagy. Indeed, inhibiting EZH2 in Hela cells induces autophagy, as demonstrated by increased LC3-II puncta (111). EZH2 knockdown and EZH2 inhibitor both increased the mRNA levels of *Tsc2*, as well as *RHOA, DEPTOR, GPI* (111). EZH2 is recruited to promoter regions of these genes *via* MTA2 (metastasis-associated member 1 family member 2) to silence their transcription, such as *Tsc2* (111). When *Tsc2* is downregulated by EZH2, the mTOR pathway is activated, leading to the inhibition of autophagy (101, 111).

There are more than 40 mammalian HATs and 18 HDACs in human genomes. Most of these HATs and HDACs have been reported to regulate autophagy in normal and diseased conditions (112, 113). In Drosophila and mice, rapamycin treatment elevated the expression of histones (H3/H4), which alters chromatin organization, induces autophagy, and mediates rapamycin-induced longevity (114). In a mouse model of stroke, attenuation of H4K16 acetylation activated the autophagic/lysosomal functions in ischemic brain and reduced the infarct volume (115).

### 3.2 Regulation of autophagy by DNA methylation

DNA methylation can inhibit autophagy through directly modifying several autophagy-related genes (*Atg*s) and silencing their transcription (**Figure 2D**). Current studies have shown that treatment of inhibitors targeting DNMTs could activate expression of certain *Atgs* and activate autophagy. In nutrient-rich environments, mTOR phosphorylates and inhibits ULK1/2, preventing the formation of autophagosomes (35, 36). When in nutrient-deprived conditions, mTOR is inhibited, which allows ULK1/2 to initiate autophagy (60). A recent study revealed that in glioblastoma, an aggressive type of brain tumor, *ULK2* gene is hypermethylated and silenced and autophagy is inhibited (116). Autophagy induced by starvation or ectopic expression of human ULK2 resulted in inhibited cancerous growth of LN229 glioma cells (116). These data suggests that transcriptional silencing of the ULK complex *via* DNA methylation modulates autophagy flux. A recent study also revealed that DNA methylation can regulate autophagy by affecting expression of LC3 and *Atg5* (61), which are crucial for the formation and elongation of autophagosomes (1, 2, 23, 46). Macrophages from aged mice exhibit lower levels of *Atg5* and LC3B, which suppressed autophagosome formation and induction of autophagy (61). Epigenetic analysis indicated that *Atg5* and LC3B genes are hypermethylated in aged macrophages, which reduced the expression of these two genes. Moreover, decreased mRNA and protein levels of *beclin1*, another upstream regulator of autophagy initiation, have been reported in many kinds of breast tumors (117). Results from sequencing of bisulfite treated DNA revealed that the CpG islands ranging from the promoter and the intron 2 of *beclin1* gene were highly methylated in certain breast cancer cells, which causes the transcriptional silencing and reduced *beclin1* expression. In addition to autophagy initiation, DNA methylation also can affect lysosomal functions through regulating expression of LAMP2, which is critical for lysosome biogenesis (59). In a patient-specific induced pluripotent stem cell (iPSCs) model of Danon disease, an X-linked disorder that leads to fatal cardiomyopathy with impaired autophagy, hypermethylation induced LAMP2 deficiency and downregulated autophagy (50). Treatment with the de-methylating agent, 5AdC could inhibit the methylation on LAMP2 genes as detected by Infinium methylation analysis, restore the expression of LAMP2, and activate autophagy, shown by decreased LC3 accumulation (50). These findings suggesting that hypermethylation of promoter and coding regions of *Atgs* reduced expression of autophagy/lysosomal related genes and inhibit autophagy.

Recent studies in neurodegenerative diseases also linked DNA methylation to autophagic deficiency (51). In Parkinson’s disease (PD), expression of autophagy/lysosomal related genes are disrupted *via* DNA methylation (51). Examining epigenetic changes in the autophagy/lysosomal pathway identified abnormal methylation on 928 cytosines, which affect expressions of 326 autophagy/lysosomal related genes in PD appendices and brains (51). ULK1, SIRT1, sphingosine kinase 1, and nicotinamide phosphoribosyltransferase, proteins that impact neurodegeneration and aging were all aberrantly methylated in PD (49, 51). In Huntington’s disease (HD), DNMT1 knockdown has been shown to strengthen autophagy and reduce Huntington (HTT)-induced neural cytotoxicity (118). Furthermore, brain-derived neurotrophic factor (BDNF) is a critical molecule involved in memory and learning, which has been shown to inhibit autophagy (24). The role of DNA methylation in regulating BDNF expression was first revealed in cultured rat neurons (119). More recent studies point to BDNF as an activator for mTOR in dendrites, as demonstrated by isolated, highly active mTOR proteins that phosphorylated eukaryotic initiation factor 4E (elF4E)-binding protein 1 (4EBP1) *in vitro* after BDNF exposure in the dendrites of cortical neurons (120). DNA methylation of BDNF promoter regions is associated with decreased levels of BDNF expression, demonstrating a link from DNA methylation to mTOR activation and suppressed autophagy (121) (**Figure 2D**).

## 4 Autophagy and neuroinflammation in neurodegenerative diseases

Autophagy has been shown to be critical in immunity, inflammation, and related diseases through controlling the burden of infectious agents, affecting differentiation of myeloid and lymphoid cells, regulating multicellular immunity, and facilitating memory responses (122). Neuroinflammation is a common pathological feature in many neurodegenerative and psychiatric disorders (123). Recent findings have linked dysregulated autophagy to the synaptic and behavioral deficits associated with neurodegenerative and psychiatric disorders, highlighting the importance of understanding the role autophagy plays in neuroinflammation (124). Accumulation of misfolded and aggravated proteins is one of the common features of most neurodegenerative diseases (3). The fundamental function of autophagy is to clear damaged organelles, protein aggregates, or lipid droplets, the accumulation of which, when autophagy is downregulated, impairs brain cell functions and induce neuroinflammation (3, 125).

In the pathophysiology of AD, dysfunctional autophagy/lysosome, inflammation with activated NLR family pyrin domain containing 3 (NLRP3) inflammasome, and protein aggregates interact together to induce the symptoms. Overexpression of Beclin1 in the hippocampus of a hAPP-transgenic mouse model of AD, activated autophagy, reduced Aβ intracellular accumulation, suppressed inflammation, and prevented neuronal death (126). Excessive p62 and LC3-positive vesicles accumulated in the brain region of temporal lobe cortex of AD patients, together with increased protein levels of LAMP1, a lysosomal maker. Further analysis indicated that these excessive autophagic vesicles are associated with activated NLRP3 inflammasome, Aβ aggregates and phosphorylated tau protein (127). Findings from a mouse line with microglia-specific *Atg7* knockout indicated that microglia cells use autophagy to clear extracellular Aβ fibrils. When autophagy is knocked down, inflammation is initiated through the Aβ-induced NLRP3/inflammasome in microglia cells (52). In PD, α-synuclein (α-syn) accumulates in neuronal and/or glial cells, and induces synaptic deficits, inflammation, and neuronal death (128). It has been shown that α-synuclein impairs autophagy *via Rab1a* (129) and genetic overexpression of TFEB and Beclin1 or administration of rapalog activated autophagy, which protected nigral neurons from α-synuclein toxicity in mouse brain (130). Neuron-released α-synuclein also induces autophagy in microglia cells *via* TLR4-NF-κB-p62 pathway and microglia used the activated autophagy to degrade α-synuclein (131). Downregulating autophagy in microglial cells in mice with over-expressed α-synuclein enhanced α-synuclein accumulation and promoted degeneration of dopaminergic neurons. On the other hand, overexpression of α-synucleinA53T mutant activated p38 kinase and subsequently inhibited the master transcriptional activator of autophagy, TFEB in microglia cells (132). p38 inhibitor SB203580 activated TFEB-mediated autophagy, suppressed NLRP3 inflammasome, prevented neuronal loss, and alleviated movement impairment in α-synucleinA53T-tg PD mice. A recent finding also indicated that in the MPTP treated PD model, autophagy deficiency in microglia exacerbates MPTP-induced NLRP3 inflammasome activity and inflammation, which caused motor dysfunction and dopaminergic neurodegeneration (133). In summary, in neurodegenerative diseases, autophagy is needed to degrade misfolded proteins and aggregates. When autophagy is impaired, inflammation-related pathways, such as NLRP3 inflammasome are activated, which induce excessive secretion of inflammatory cytokines and occurrence of neuroinflammation (134).

### 4.1 Autophagy and neuroinflammation in glia cells

**Figure 3:**
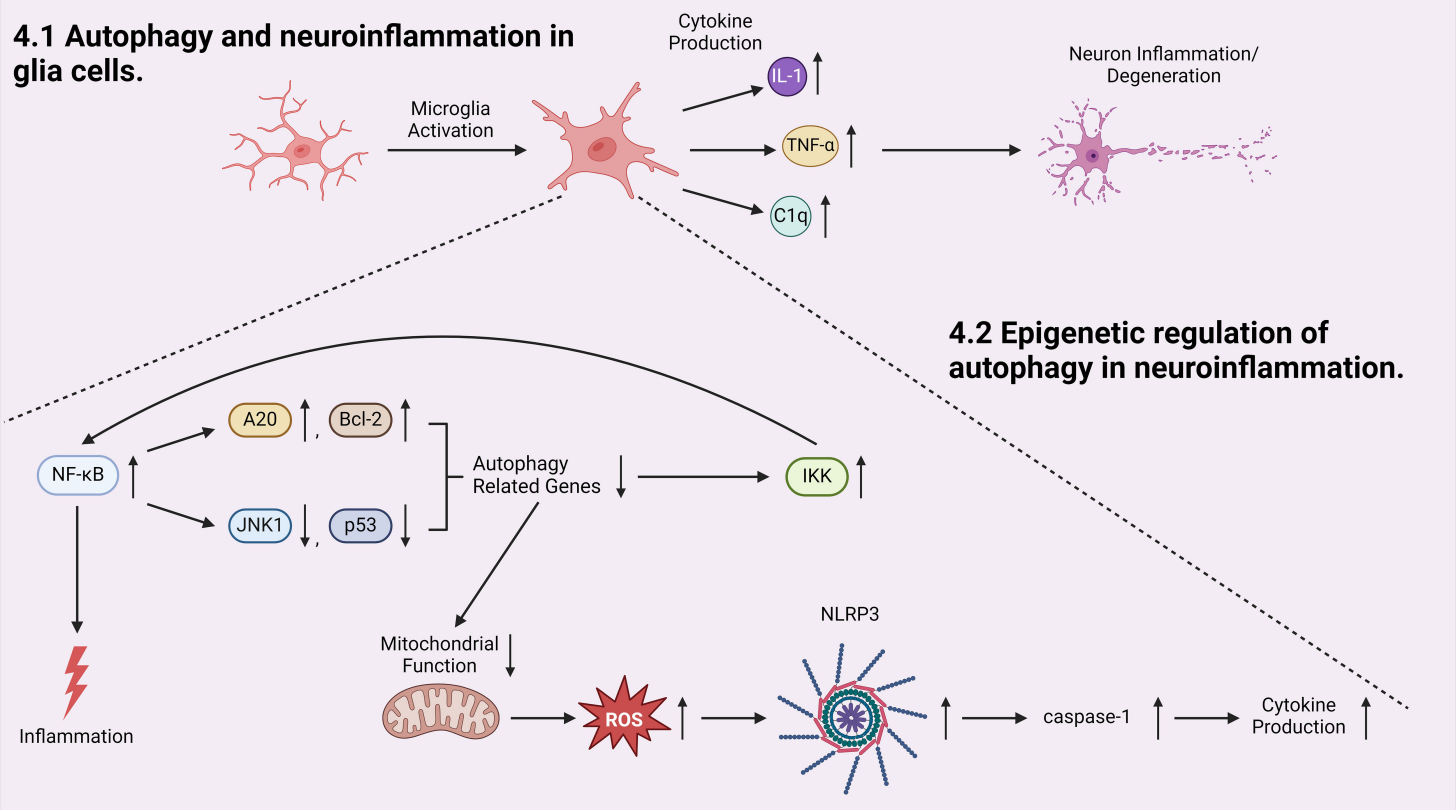
Autophagy is involved in the initiation and progression of neuroinflammation. Microglia cell activation produces pro-inflammatory cytokines which initiates neuron inflammation and degeneration. Inside the activated microglia cell, transcriptional factor NF-κB, an activator of inflammation, induces a positive-feedback loop in which NF-κB promotes the production of autophagy inhibitors A20 and Bcl-2 and inhibits the production of autophagy inducers: JNK1 and p53. Both pathways result in decreased expression of autophagy-related genes, which downregulates autophagic degradation and stabilizes IKK, elevating the activity of NF-κB. At the same time, when autophagy is inhibited, mitochondrial function is dysregulated, generating more reactive oxygen species (ROS) which leads to NLRP3/inflammasome and subsequent caspases-1 activation, and induces elevated cytokine production.

The discussion above has pointed out that glia cells play important roles in development of autophagy-associated neuroinflammation. Microglia are the primary immune cells in the CNS that regulate brain development, maintenance of neural pathways, and injury repair (135). Microglia cells play a vital role in neuroinflammation by secreting key inflammatory mediators, such as interleukin-1β (IL-1 β) and tumor necrosis factor-α (TNF-α) (136) (**Figure 3**). While the release of these factors is intended to prevent further tissue damage, they are also toxic to neurons, and chronic microglial activation is one of the major contributing factors to the pathogenesis of neurodegenerative disorders (54, 137). For example, activated microglia has been shown to play a crucial role in the pathogenesis of multiple sclerosis (MS) (54–57). Microglia associated inflammation within CNS results in the demyelination of neurons in MS patients (54, 57). Selective deletion of *Pik3c3*, *Atg5*, or *Atg7* in myeloid cells resulted in attenuated severity of experimental autoimmune encephalomyelitis (EAE), the prototypic animal model of MS (57). Recent studies on infiltrated T cells in brains of patients with MS revealed elevated ATG5 expression compared to non-diseased controls. Transcriptome data also showed that ULK1 was upregulated (57). These findings suggest a strong connection between dysregulated autophagy and neuroinflammation in activated microglia and other inflammatory cells in MS. Additionally, dysregulated mitochondrial function in activated glia cells leads to elevated production and accumulation of reactive oxygen species (ROS), which activates the NLRP3 inflammasome pathway, leading to the activation of caspases-1 and the production of inflammatory cytokines (138) (**Figure 3**).

Furthermore, autophagy is able to regulate microglial activation by regulating the expression of inflammatory cytokines and modulating the activity of inflammation-related transcription factors (139). Transcription factor nuclear factor κB (NF-κB) regulates multiple adaptive immune functions and inflammatory responses and is involved in the production of pro-inflammatory cytokines (140) (**Figure 3**). Recently, accumulating evidence pointed towards the reciprocal crosstalk between NF-κB signaling and autophagy (141, 142). Inhibitor of IKK/NF-κB signaling promotes autophagy by enhancing the expression of genes involved in autophagosome formation, including *beclin1*, BAG3-HspB8 complex, *Atg5*, and LC3 (141, 143, 144). Conversely, activated NF-κB inhibits autophagy by increasing the expression of autophagy inhibitors such as A20 and Bcl-2, or suppressing the expression of autophagy inducers such as JNK1 and p53 (141, 145–147). In parallel, autophagy has been shown to regulate NF-κB pathway by degrading IKK components and NF-κB-inducing kinases, again demonstrating a reciprocal regulatory relationship between the autophagy/lysosomal pathway and IKK/NF-κB (141). Moreover, a recently established cargo-selective autophagic process termed “synucleinphagy” has been observed to promote neuroprotection by clearing neuron-derived α-synuclein in PD mouse models (131). Aggregated α-synuclein is a major component of Lewy bodies which are associated with neurodegenerative diseases including PD and Lewy body dementia (131). TLR4-NF-κB signaling *via* transcriptional regulation of the autophagy adaptor, p62/SQSTM1, mediates this process, which results in the absorption of external α-synuclein (131). Many studies have shown that activated autophagy suppresses inflammation in microglia cells through inhibiting NLRP3 inflammasome pathway in research models of neurodegenerative diseases (148, 149).

Astrocytes are key regulators of neuronal homeostasis within the CNS (150). Inflammatory (A1) and neuroprotective (A2) astrocytes were identified in initial studies examining astrocyte responses to stress (151, 152). Activated microglia induce the transformation of naïve astrocytes into A1 astrocytes by releasing IL-1α, TNFα, and C1q cytokines, each of which is essential for inducing A1 astrocytes (151, 152). A1 astrocytes lose many normal functions, such as the promotion of neuronal survival, which causes fewer and weaker synapses (151). A2 astrocytes are neuroprotective and marked with increased expression of anti-inflammatory cytokine TGFβ, which contributes to synaptogenesis (151). TGFβ prevents apoptotic cell death in neurons by inhibiting caspase-3 activation, and current studies point to a neuroprotective role for TGFβ against Aβ toxicity in AD models both *in vitro* and *in vivo* (153). By regulating the activation of microglial cells *via* ROS and IKK/NF-κB pathways, autophagy can prevent the formation of the inflammatory A1 astrocytes by decreasing the release of pro-inflammatory cytokines that are associated with the transformation of naïve into A1 astrocytes (139, 151).

### 4.2 Epigenetic regulation of autophagy in neuroinflammation

Epigenetic regulation such as histone acetylation can directly affect the interaction between autophagy and the inflammasome to regulate the process of neuroinflammation. In an animal model of sevoflurane-induced cognitive impairment in aged mice, histone acetylation and autophagy are downregulated and NLRP3 inflammasome is activated (154). Treatment of suberoylanilide hydroxamic acid (SAHA), an inhibitor of HDACs, to these mice protects hippocampal neurons and improves cognitive function through restoring impaired autophagy and inhibiting NLRP3 inflammasome activation. SAHA increases histone H3 and H4 acetylation, which subsequently activates autophagy. Importantly, autophagy inhibitor 3-MA abolishes the inhibitory effect of SAHA on neuroinflammation indicating that autophagy activation plays a key role in the mechanism by which histone acetylation suppresses neuroinflammation. In another research, intrathecal injection of calcitonin gene-related peptide (CGRP) and chronic constriction injury (CCI), animal models of nerve injury and neuropathic pain, increase acetylation of H3K9 in astrocytes located in the spinal dorsal horn of rats (155). ChIP-seq data revealed that CGRP treatment in astroglia cells altered H3K9ac enrichment on promoters of genes involved in proliferation, autophagy, and inflammation. Administration of H3K9 acetylation inhibitor, anacardic acid (AA) in CGPR treated astroglia cells blocks the CGPR-induced overexpression of the autophagy and inflammation related genes. This study identified a set of autophagy-related genes and inflammatory genes separately as direct targets activated by acetylation of H3K9, however, whether autophagy is causally related to neuroinflammation regulation by histone acetylation needs to be further examined. More recently, a study discovered a new HDAC11 specific inhibitor named compound five, “5” (156). 5 inhibits deacetylase function of HDAC11 and attenuate microglia-medicated neuroinflammation through induction of autophagy and reduction of reactive nitrogen species. 5 further shows anti-depressant activity by inhibiting microglia activation in LPS-treated mice. It is significant in that this study is the first to show a therapeutic potential of specific pharmacological inhibitor of HDAC11 for depressive disorders.

## 5 Implications in neuroinflammatory-induced diseases

Synaptic deficits are associated with many kinds of neurological diseases. For example, it has been revealed that reduced synapse density in hippocampal neurons critically contributes to the cognitive deficits associated with AD. At the same time, multiple studies show that neuroinflammation is present in a variety of neurodegenerative diseases, psychiatric disorders, and ASDs, and glia cells may play a central role in mediating neuroinflammation. Dysregulated glia cells induce inflammation in the brain and affect synapse morphology, density, and plasticity. When investigating new treatments for neurological disorders, more and more studies are focusing on targeting pro-inflammatory pathways. Epigenetic regulation of autophagy to ameliorate neuroinflammation presents a novel and promising therapeutic strategy in treating synaptic and behavioral deficits in neurological diseases. Further research on the interactions between autophagy/lysosomal pathways, neuroinflammation, and epigenetic regulation of these two processes could provide valuable insights for the development of future therapeutic and pharmacological treatments for diseases associated with neuroinflammation.

## Author contributions

IB: Writing – original draft, Writing – review & editing. CK: Writing – original draft, Writing – review & editing. ZZ: Writing – review & editing. BR: Writing – review & editing. J-YH: Writing – review & editing. RZ: Writing – review & editing. JY: Writing – original draft, Writing – review & editing.
